# *Clonorchis sinensis* and cholangiocarcinoma: molecular mechanisms and biomarker advances

**DOI:** 10.1051/parasite/2026011

**Published:** 2026-04-01

**Authors:** Chenlin Huang, Yinjuan Wu, Aoxun Wu, Linya Huang, Shu Fang, Chunyan Xu, Tianhao Liu, Yichen Li, Xuerong Li

**Affiliations:** 1 Department of Parasitology, Zhongshan School of Medicine, Sun Yat-sen University Guangzhou China; 2 Department of Basic Medicine, Zhongshan School of Medicine, Sun Yat-sen University Guangzhou China; 3 Basic Medical Experimental Teaching Center, Zhongshan School of Medicine, Sun Yat-sen University Guangzhou China; 4 Key Laboratory for Tropical Diseases Control, Ministry of Education, Sun Yat-sen University Guangzhou China; 5 Provincial Engineering Technology Research Center for Biological Vector Control Guangzhou China; 6 Department of Clinical Medicine, Zhongshan School of Medicine, Sun Yat-sen University Guangzhou China

**Keywords:** *Clonorchis sinensis*, Molecular Biomarkers, Cholangiocarcinoma, Carcinogenic Mechanisms, Preneoplastic Lesions

## Abstract

*Clonorchis sinensis*, a common food-borne liver fluke in East Asia, is a Group 1 carcinogen strongly linked to cholangiocarcinoma. In recent years, molecular biology and multi-omics studies have revealed that this parasite drives chronic inflammation of the bile duct epithelium, epigenetic abnormalities, and the formation of precancerous lesions. Concurrently, circulating miRNAs, DNA methylation patterns, differential protein expression, metabolite profiles, and parasite-specific antigens have been proposed as potential early molecular biomarkers, which offers new avenues for the non-invasive detection of precancerous conditions. However, current research mainly remains at the laboratory stage and studies have small-scale cohorts, lacking multi-center, large-sample prospective validation and standardized detection protocols, which limits their clinical applicability. Furthermore, traditional imaging and histological methods exhibit limited sensitivity for early identification. This review aims to systematically summarize the molecular carcinogenic mechanisms associated with *C. sinensis* infection, recent advances in molecular biomarker research, and strategies for identifying precancerous lesions. It will particularly focus on discussing the major obstacles in clinical translation and future directions, with the goal of providing insights for early screening and prevention strategies.

## Abstract (fr)

*Clonorchis sinensis*, une douve du foie fréquemment transmise par voie alimentaire en Asie de l’Est, est un cancérogène du groupe 1 fortement associé au cholangiocarcinome. Ces dernières années, des études de biologie moléculaire et multi-omiques ont révélé que ce parasite induit une inflammation chronique de l’épithélium des voies biliaires, des anomalies épigénétiques et la formation de lésions précancéreuses. Parallèlement, les microARN circulants, les profils de méthylation de l’ADN, l’expression différentielle des protéines, les profils métaboliques et les antigènes spécifiques du parasite ont été proposés comme biomarqueurs moléculaires précoces potentiels, ouvrant ainsi de nouvelles perspectives pour la détection non invasive des états précancéreux. Cependant, les recherches actuelles restent principalement au stade du laboratoire et portent sur de petites cohortes, sans validation prospective multicentrique à grande échelle ni protocoles de détection standardisés, ce qui limite leur applicabilité clinique. De plus, les méthodes d’imagerie et histologiques traditionnelles présentent une sensibilité limitée pour le diagnostic précoce. Cette revue vise à synthétiser de manière systématique les mécanismes moléculaires de la carcinogenèse associés à l’infection par *C. sinensis*, les avancées récentes en matière de recherche sur les biomarqueurs moléculaires et les stratégies d’identification des lésions précancéreuses. Elle s’attache plus particulièrement à examiner les principaux obstacles à la transposition clinique et les perspectives d’avenir, afin d’éclairer les stratégies de dépistage précoce et de prévention.

## Introduction

**Figure 1:**
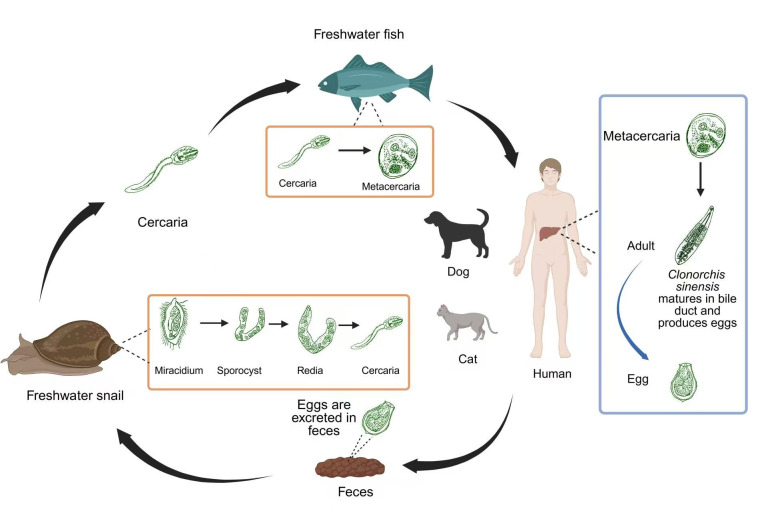
Life cycle of *Clonorchis sinensis* and its major endemic regions. Adult flukes in mammalian hosts release eggs *via* feces. Eggs hatch in snails, develop into cercariae, which encyst as metacercariae in freshwater fish. Human infection occurs through consumption of raw fish. Major endemic areas include China (mainly in Jilin, Guangdong, Guangxi and Heilongjiang), Korea, Vietnam, Eastern Russia and Japan. The red areas (shading) represent the regions endemic for *Clonorchis sinensis*.

*Clonorchis sinensis* is a food-borne trematode widely distributed in East Asia, particularly in China, the Korean Peninsula, northern Vietnam, and the Far East of Russia. The life cycle of *C. sinensis* involves two intermediate hosts and one definitive host. Adult worms reside in the intrahepatic bile ducts of humans and other mammals and release eggs that are excreted in feces. Eggs are ingested by freshwater snails, where miracidia hatch and develop through sporocyst and redia stages to cercariae. Cercariae infect freshwater fish and encyst as metacercariae. Humans acquire infection by consuming raw or undercooked fish containing metacercariae, which excyst and migrate to the bile ducts to mature into adult worms (Fig. 1) [68].

In China, the main endemic regions are found along the Songhua and Nenjiang Rivers in the northeast, and the Pearl River basin in the south. Based on surveillance data from ~1.87 million individuals across 31 Chinese provinces, including annual sampling in ~300 counties (fresh stool specimens tested for *C. sinensis* eggs *via* the Kato-Katz method) and Bayesian geostatistical modeling integrated with population weighting, the current estimated number of *C. sinensis*-infected individuals in China is ~9.46 million. The vast majority of these cases are concentrated in Jilin (~0.71%, ~0.19 million), Heilongjiang (~1.29%, ~0.51 million), Guangdong (~2.99%, ~3.71 million), and Guangxi (~8.92%, ~4.2 million) provinces (Fig. 1) [57]. In the Republic of Korea, the number of infected individuals reached several million during the 1970s and 1980s, with local prevalence rates exceeding 20%. Through subsequent large-scale screening and population-based anthelmintic treatment, the prevalence has recently declined to below 5%. In contrast, some provinces in northern Vietnam still report high endemicity levels exceeding 20%. This comparison indicates that interventions against liver flukes are of great significance in public health. The infection rate and endemic intensity of *C. sinensis* has shown marked variation across time and regions. However, the transmission route of *C. sinensis* is largely consistent across different regions and is primarily driven by the consumption of raw or undercooked freshwater fish, with no substantial regional variation in the infection pathway. The parasite is capable of surviving in the human biliary system for decades, resulting in higher infection rates among the elderly and a greater spawning rate. In addition, infection intensity in highly endemic communities often differs greatly from national averages. As a result, large-scale estimations may not fully reflect the actual local disease burden [28].

Epidemiological and experimental data indicate a significant correlation between *C. sinensis* infection and the development of cholangiocarcinoma (CCA). The International Agency for Research on Cancer (IARC) has classified infections with *C. sinensis* and *Opisthorchis viverrini* (*O. viverrini*) as Group 1 carcinogens [25], based on sufficient epidemiological evidence demonstrating a significant association with CCA risk. This is supported by case-control studies reporting adjusted ORs of 2.7 to 13.6 [25, 31, 56].

Mechanistic studies reveal that liver flukes residing in the bile ducts induce chronic inflammation, oxidative/nitrative stress, and endogenous nitrosamine production. Furthermore, parasite secretions promote abnormal epithelial cell proliferation and epigenetic alterations, ultimately leading to carcinogenesis [28]. *Clonorchis sinensis* causes continuous injury and repair in biliary epithelial cells through mechanical stimulation, ESPs, and the induction of chronic host inflammatory responses, thereby establishing the biological foundation for preneoplastic lesions and eventual malignant transformation [68, 70].

Advancements in molecular biology and omics technologies (including transcriptomics, proteomics, metabolomics, and exosome/circulating nucleic acid analysis) have begun to elucidate the roles of numerous molecules secreted by *C. sinensis*. Experimental studies have demonstrated that *C. sinensis* excretory/secretory products (ESPs) and extracellular vesicles (EVs) can induce the secretion of pro-inflammatory cytokines (*e.g.*, IL-6, TNF-α) by cholangiocytes and activate pathways such as EGFR→RAS/MAPK/ERK, NF-κB, PI3K/Akt, thereby stimulating the carcinogenic process [50, 51, 75, 80, 83]. Additionally, specific molecules like legumain and cathepsin B have been shown to promote the migration of CCA cells and tissue damage. These findings highlight parasite-host molecular interactions as key drivers of preneoplastic changes [1, 11, 14].

In clinical practice, the early detection of *C. sinensis*-associated preneoplastic lesions faces multiple challenges. Traditional imaging and endoscopic techniques have limited sensitivity for identifying early or subtle epithelial changes, while conventional cytology or histology sampling is susceptible to false negatives due to sampling location and tumor heterogeneity [31]. In recent years, research on early molecular biomarkers has focused on circulating miRNAs, exosomal components, DNA methylation profiles, differentially expressed proteins (including parasite-derived antigens), and metabolite profiles. In contrast to conventional tissue-based biopsy, these liquid biopsy approaches show potential for non-invasive or minimally invasive early screening. Nevertheless, most candidate biomarkers for liquid biopsy are still under preliminary evaluation in small-scale cohort or experimental validation, lacking multi-center, large-sample prospective validation, and methodological standardization [2, 60, 62, 66].

Therefore, this review aims to provide a comprehensive overview of recent progress along three main themes: i) mechanisms by which *C. sinensis* promotes CCA, ii) molecular/omics biomarkers for the detection of preneoplastic lesions, and iii) strategies for precancerous lesion identification. It will comprehensively summarize current findings on parasite-derived oncogenic molecules, the regulation of host signaling pathways by parasite EVs/ESPs, host-level epigenetic changes (DNA methylation) and circulating small RNA evidence, as well as advances in proteomics and metabolomics for biomarker discovery. This review will also address the technical, epidemiological, and implementation challenges associated with the clinical translation of these biomarkers, aiming to provide a theoretical and methodological reference for future strategies in early screening, disease progression monitoring, and intervention [81, 93].

## The mechanisms of *C. sinensis* infection and the evolution of preneoplastic lesions

Infection with *C. sinensis* is recognized by the World Health Organization as a definitive human carcinogen, closely related with the development of CCA [31, 73]. Due to long-term residence of the parasite within the biliary system, its metabolic products will arouse host immune response, which contribute to a continuous process of inflammation-damage-repair-malignant transformation [33].

Genomic and transcriptomic studies have revealed that *C. sinensis* possesses rich secretory proteins and regulatory molecules, many of which are implicated in host immunomodulation and carcinogenesis [23, 78, 90, 91]. Its ESPs and EVs can directly act on cholangiocytes, activating multiple signaling pathways including ERK, NF-κB, and FAK/Src, and subsequently promote cell proliferation, migration, and invasion [48, 50, 83, 84].

### Carcinogenic mechanisms of *C. sinensis* ESPs

**Figure 2:**
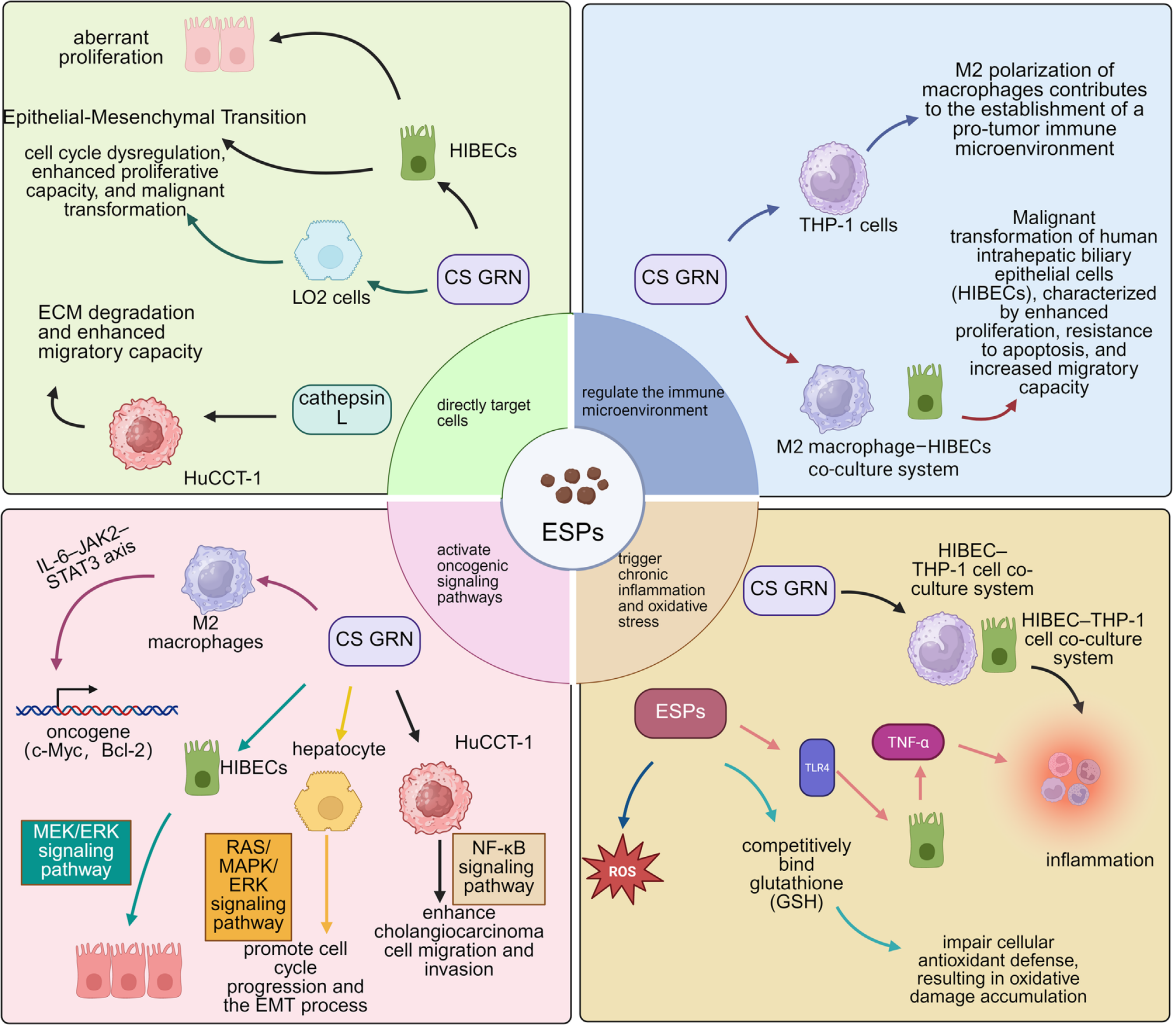
Pathogenic mechanisms induced by *C. sinensis* excretory/secretory products (ESPs). It illustrates the mechanisms by which ESPs promote biliary epithelial cell proliferation, fibrosis, and malignant transformation through the activation of pathways such as EGFR, ROS, and inflammatory signaling. HIBECs refers to Human intrahepatic biliary epithelial cells. IL-6: Interleukin-6, JAK2: Janus Kinase 2, STAT3: Signal Transducer and Activator of Transcription 3, TGF-β: Transforming Growth Factor-beta, TNF-α: Tumor Necrosis Factor-alpha, HIBECs: Human In Biliary Epithelial Cells, THP-1: Human monocytic leukemia cell line.

The carcinogenic potential of *C. sinensis* ESPs centers around *C. sinensis* Granulin (*Cs*GRN), which serves as a key nexus linking parasitic infection to CCA and hepatocellular carcinoma (HCC) by directly inducing hepatocyte proliferation and EMT, regulating macrophage M2 polarization, and activating the MEK/ERK-STAT3 pathway. Molecules regulating MMP-9 enhance tumor invasiveness *via* the ERK1/2-NF-κB pathway, acting as major drivers of tumor progression. Other molecules, such as *Cs*-GT and *Cs*-severin, primarily exhibit anti-inflammatory functions; however, their role in regulating oxidative stress during chronic infection may indirectly foster a procarcinogenic microenvironment. These three kinds of molecules act cohesively, constituting the procarcinogenic network associated with *C. sinensis* infection [19, 30, 50, 75]. Recent studies further identified that specific molecules within *C. sinensis* ESPs, such as legumain, can enhance the metastatic potential of CCA cells [14] (Fig. 2).

*Clonorchis sinensis* ESPs can activate the EGFR. In CCA, approximately 20% of cases have FGFR2 fusions or mutations, which drive constitutive activation of pathways like RAS–MAPK/ERK, promoting tumorigenesis and progression. Although FGFR2 inhibitors are approved for such patients, monotherapy efficacy is often limited due to feedback activation of EGFR signaling, which leads to drug resistance or partial response. Research has also identified significant signaling crosstalk between EGFR and FGFR2. Combined inhibition of both receptors can block this feedback, sustain suppression of MEK/ERK and mTOR signaling, and induce apoptosis, thereby significantly enhancing treatment efficacy and delaying resistance. This suggests that *C. sinensis*, *via* its ESPs activating EGFR, may exert molecular synergy with FGFR2 mutation-driven CCA [85].

Chronic inflammation is a critical driver of preneoplastic lesions. *Clonorchis sinensis* ESPs can also stimulate cholangiocytes *via* TLR4 to secrete TNF-α, causing local inflammation and tissue damage [88]. This persistent immune response in the biliary microenvironment drives oxidative stress and DNA damage, resulting in epithelial hyperplasia and dysplasia [31].

In mouse models, *C. sinensis* infection (and its ESPs) combined with exposure to N-nitroso compounds (*e.g.*, NDMA) promotes periductal fibrosis by activating NADPH oxidase (NOX); this will result in excessive reactive oxygen species (ROS) production and subsequent upregulation of fibrotic proteins like fibronectin and collagen type I. Mouse studies show that the intensity of the immune response to NOX subunits (p47phox, p67phox) and the deposition of collagen and fibronectin positively correlate with infection duration, NDMA exposure, and lesion severity. These pathological changes can be alleviated by NOX inhibitors (*e.g.*, DPI) or antioxidants (*e.g.*, NAC) [26].

Regarding antioxidant mechanisms, *Cs*-GT within *C. sinensis* ESPs can competitively bind glutathione (GSH) – a key scavenger of ROS, reducing cellular antioxidant capacity and allowing oxidative damage to accumulate [30]. Chronic oxidative stress can lead to mutations in tumor suppressor genes like p53, while simultaneously activating procarcinogenic pathways such as NF-κB, thereby accelerating malignant transformation. At the same time, studies in hamster models have shown that antioxidants like resveratrol (RSV) or SkQ1 significantly reduce hepatobiliary pathologies induced by *Opisthorchis felineus* infection, decreasing bile duct hyperplasia, proliferation, fibrosis, and the accumulation of lipids and glycogen. They also downregulate the lipid peroxidation product 4-hydroxynonenal, alleviating host oxidative stress and mitigating the adverse effects of praziquantel (PZQ) on the liver parenchyma [92]. These findings together underscore the pivotal role of oxidative stress in carcinogenesis.

Remarkably, the carcinogenic mechanisms of another dangerous liver fluke *O. viverrini* are highly similar to *C. sinensis.* The species *O. viverrini* also secrete a granulin-like growth factor (*Ov*GRN) which stimulates cholangiocyte proliferation, migration, and wound repair responses *via* activation of the EGFR/MAPK pathway, paralleling the functions of the *Cs*GRN. In addition, *Ov*-derived cysteine proteases and detoxification enzymes, including glutathione S-transferases and thioredoxin, promote extracellular matrix remodeling, oxidative stress modulation, and chronic inflammation. These ESP-mediated processes activate NF-κB signaling pathways and will lead to sustained inflammatory responses, epithelial hyperplasia, and fibrosis. The close mechanistic conservation between *O. viverrini* and *C. sinensis* emphasizes the shared molecular basis of liver fluke-associated cholangiocarcinogenesis [68].

### Carcinogenic mechanisms of *C. sinensis* EVs

Recent studies have demonstrated that *C. sinensis* mediates cross-species communication through both ESPs and EVs, which contain bioactive proteins, lipids, and non-coding RNAs. They can be taken up by host cells, thereby modulating cellular signaling pathways, immune responses, and the tumor microenvironment [51, 93].

#### EVs promoting the proliferation and migration of CCA cells

Pan *et al.* were the first to systematically reveal the role of *C. sinensis*-derived EVs (*Cs*EVs) in promoting malignant phenotypes in CCA cells [51]. Their study demonstrated that *Cs*EVs are effectively internalized by human CCA cells (RBE, HuCCT1) and subsequently significantly promote cell proliferation, clonogenicity, migration, and invasion. Mechanistically, *Cs*EVs activate the NF-κB and ERK signaling pathways, inducing EMT, characterized by decreased E-cadherin and increased N-cadherin and Vimentin expression. This procarcinogenic process mediated by *Cs*EVs could be partially reversed using the NF-κB inhibitor BAY 11-7082, confirming the core role of the NF-κB/EMT axis. These findings suggest that *Cs*EVs can reprogram cholangiocyte signaling pathways, thereby driving the early formation and progression of CCA.

#### The carcinogenic role of miRNAs carried by *Cs*EVs

MiRNAs within EVs are key functional molecules. Through high-throughput sequencing and functional validation, studies have identified that various parasite-derived miRNAs carried by *Cs*EVs can directly regulate host immune responses and cellular metabolic states.

Zhang *et al.* reported that Csi-let-7a-5p in *Cs*EVs can be taken up by macrophages and target Clec7a and SOCS1, which will subsequently activate the NF-κB signaling pathway and induce M1 pro-inflammatory macrophage polarization, leading to biliary epithelial damage and fibrosis [93]. In mouse models, using *Cs*EVs with inhibited Csi-let-7a-5p significantly alleviated cholangitis and biliary injury, indicating that Csi-let-7a-5p is an important molecule mediating the inflammation-carcinogenesis cascade. Another study found that Csi-miR-96-5p promotes ferroptosis-related metabolic dysregulation in cholangiocytes by regulating the PTEN/SLC7A11/GPX4 axis, thus enhancing cell proliferation and migration capabilities [51]. These results confirm that miRNAs in *Cs*EVs act not only as immunoregulatory factors but also as “cross-species carriers” of procarcinogenic signals.

Exosomal miR-21 exhibits consistent upregulation across various cancer types and is considered a “pan-cancer” oncogenic miRNA. It enhances cell proliferation and anti-apoptotic capacity by suppressing tumor suppressor genes like PTEN and PDCD4 [39]. Although a direct homolog of miR-21 has not yet been reported in *C. sinensis*, given its broad evolutionary conservation and oncogenic characteristics, similar mechanisms are hypothesized to potentially exist in *Cs*EVs-host interactions.

#### EV-mediated remodeling of the inflammatory microenvironment

**Figure 3:**
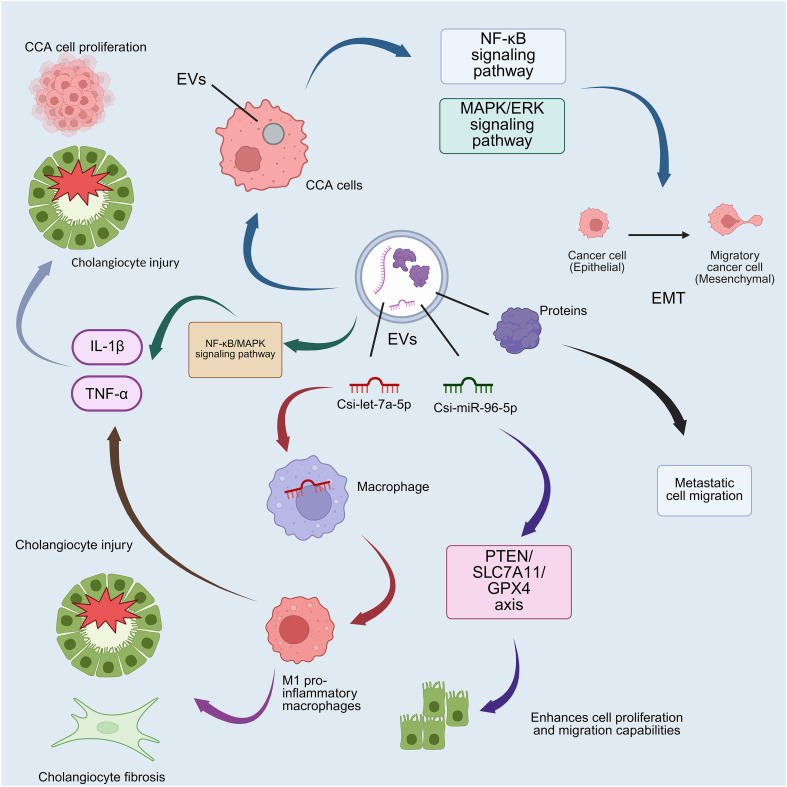
Pathogenic mechanisms induced by *Cs*EVs. It depicts how *Cs*EVs facilitate processes such as cholangiocarcinoma cell proliferation, migration, and inflammation microenvironment formation by transferring bioactive molecules such as miRNAs, proteins and activating pathways like NF-κB/EMT. CCAs: Cholangiocarcinoma, IL-1β: Interleukin-1 beta, TNF-α: Tumor Necrosis Factor-alpha, EVs: Extracellular Vesicles, NF-κB: Nuclear Factor kappa-light-chain-enhancer of activated B cells, ERK: Extracellular Signal-Regulated Kinase, EMT: Epithelial-Mesenchymal Transition, PTEN: Phosphatase and Tensin Homolog, SLC7A11: Solute Carrier Family 7 Member 11, GPX4: Glutathione Peroxidase 4.

Beyond their direct effects on cholangiocytes, *Cs*EVs can also modulate host immune cell functions, fostering a chronic inflammatory microenvironment. Experiments show that M1 macrophages stimulated by *Cs*EVs secrete high levels of TNF-α and IL-1β, inducing biliary epithelial cell damage and periductal inflammatory infiltration [93]. *Cs*EVs can also activate the Toll-like receptor (TLR)-ERK pathway in cholangiocytes, promoting the secretion of IL-6 and TNF-α, which further amplifies the inflammatory response and tumor cell growth [80]. This immune-signaling positive feedback loop creates conditions for sustained inflammation within the tumor microenvironment and the malignant transformation of epithelial cells (Fig. 3).

#### Auxiliary carcinogenic effects of protein components

Proteomic analysis of *Cs*EVs has revealed an enrichment of various functional proteins, such as heat shock proteins (HSPs), annexins, and enolases [51]. These molecules are able to modulate cellular stress responses, energy metabolism, and cytoskeletal remodeling, indirectly promoting cancer cell migration and resistance to apoptosis. What’s more, some parasite-derived proteins may mimic host ligands, activating MAPK or PI3K/AKT signaling pathways, thereby synergistically facilitating the development of CCA.

The carcinogenic mechanisms mediated by EVs also exhibit significant conservation between *C. sinensis* and *O. viverrini.* Both flukes utilize EVs as critical intercellular messengers. Their surface tetraspanins (TSPs) are essential for EVs biogenesis and subsequent clathrin-dependent endocytosis by human cholangiocytes. Compared with the tetraspanins of *C. sinensis*, the research on TSPs from *O. viverrini* is more systematic and in-depth, with clearer functional characterization and more comprehensive mechanistic elucidation. *Ov*EVs also contain miRNAs predicted to target host genes involved in growth factor signaling, inflammation, and angiogenesis. This further indicates that the delivery of regulatory RNA molecules *via* EVs is employed to reshape the host microenvironment [10, 51, 54].

### Other carcinogenic mechanisms

Epigenetic alterations, such as DNA hypermethylation, also play a vital role in cholangiocarcinogenesis and may be induced by chronic parasitic infection [6, 65].

The persistent interplay of these molecular and immune mechanisms progressively drives the evolution of biliary epithelium from inflammatory changes to preneoplastic lesions. Some studies have summarized molecular biomarkers applicable for the early identification of CCA, including molecules associated with pathways involving MMP-9, Integrin β4, and IL-6, among others [48, 53]. Consequently, *C. sinensis* infection is not only a direct etiological factor for CCA, but also provides a critical window for investigating preneoplastic molecular biomarkers [31, 73].

## Comparative analysis of ESP and EV components with *O. viverrini*

A comparative analysis may help to clarify shared and unique carcinogenic pathways for liver flukes and formulate control strategies. In *O. viverrini*, ESPs similarly serve as core mediators triggering host cell carcinogenesis. We analyze the composition of ESPs from both *C. sinensis* and *O. viverrini* below.

Regarding composition, the ESPs of *O. viverrini* and *C. sinensis* share considerable similarities. Both contain growth factor-like proteins (*Cs*GRN/*Ov*-GRN-1), cysteine proteases, detoxifying enzymes like GSH transferases (GST), metabolism-related enzymes, and immunologically active proteins [9, 32]. The cysteine proteases can degrade host tissue components and disrupt immune cells; the detoxifying enzymes protect the parasite from oxidative damage; the metabolism-related enzymes sustain parasite energy metabolism; and the immunologically active proteins interfere with host immune responses, facilitating parasite survival.

However, differences still exist. For instance, *O. viverrini* ESPs contain unique components such as thioredoxin (*Ov*-Trx-1), which modulates host redox balance and promotes cholangiocyte survival, and a novel 30 kDa protein with potential proliferative effects [16, 42]. In contrast, a distinctive immunodiagnostic protein in *C. sinensis* ESPs is legumain [14], which primarily serves as a serodiagnostic antigen aiding disease diagnosis, with its direct role in regulating cell proliferation not explicitly established. Additionally, *Cs*-severin has been reported to inhibit inflammatory responses (reducing secretion of IL-17A and GM-CSF) and alleviate symptoms in rheumatoid arthritis (RA) and ankylosing spondylitis (AS) [30].

**Figure 4:**
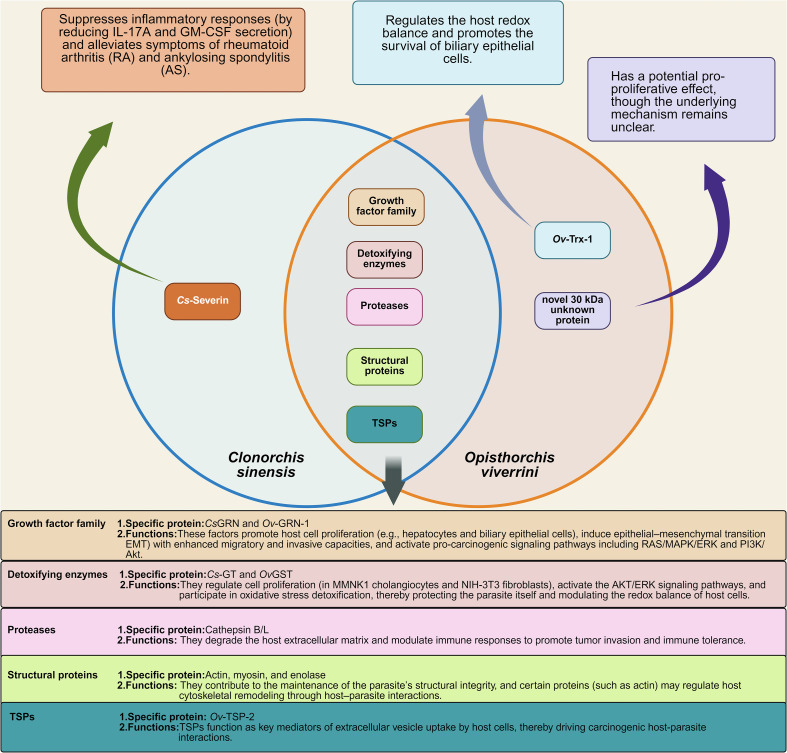
Similarities and differences in ESPs between *O. viverrini* and *C. sinensis.* It compares the shared and unique components in the ESPs of *O. viverrini* and *C. sinensis*, including growth factor-like proteins, cysteine proteases, and immunomodulatory proteins, highlighting their potential differential roles in carcinogenesis. IL-17A: Interleukin-17A, GM-CSF: Granulocyte-Macrophage Colony-Stimulating Factor, RA: Rheumatoid Arthritis, AS: Ankylosing Spondylitis, TSPs: Tetraspanins, *Ov*-Trx-1: *O. viverrini* Thioredoxin-1, *Cs*GRN: *Clonorchis sinensis* Granulin, *Ov*-GRN-1: *O. viverrini* Granulin-1, *Cs*-GT: *Clonorchis sinensis* Glutathione Transferase, *Ov*GST: *O. viverrini* Glutathione S-Transferase, MMNK1: Human cholangiocyte cell line, NIH-3T3: Mouse embryonic fibroblast cell line, AKT: Protein Kinase B, RAS: Rat Sarcoma virus gene, MAPK: Mitogen-Activated Protein Kinase, PI3K: Phosphoinositide 3-Kinase, *Ov*-TSP-2: *O. viverrini* Tetraspanin-2.

In terms of proteomic characteristics, research on *O. viverrini* has more clearly defined the molecular structures and targets of some carcinogenesis-related proteins. In contrast, studies on *C. sinensis* have placed greater emphasis on enzymatic activity analysis, with relatively less focus on the identification of specific procarcinogenic proteins (Fig. 4) [50].

Beyond ESPs, both *O. viverrini* and *C. sinensis* secrete EVs that mediate critical host-parasite interactions. Notably, TSPs play pivotal roles in EV biogenesis, cargo sorting, and cellular uptake. In *O. viverrini*, *Ov*-TSP-2 (a CD63-family tetraspanin) is abundantly expressed on the tegument and EV membranes and has emerged as a promising vaccine target. Disruption of *Ov-tsp-2* using CRISPR–Cas9 leads to tegumental damage, substantially decreases EV release, and attenuates EV internalization by cholangiocytes, emphasizing its indispensable role in EV-driven pathogenic signaling [10, 54].

Although tetraspanins have been detected in *C. sinensis*, their subtype diversity and mechanistic roles have yet to be systematically elucidated, providing an avenue for future investigation [51].

By comparing ESP and EV components of these two dangerous liver-flukes, we can gain insights into the common mechanisms of fluke-induced cancer and this will guide the development of comprehensive control strategies.

## Advances in molecular biomarker research

We first clarified carcinogenic mechanisms (*via* ESPs, EVs and key molecules), as mechanisms reveal critical molecules that lay the foundation for biomarker research. The development of molecular biology and omics technologies has provided increasing evidence that molecular products generated during *C. sinensis* infection, as well as host response molecules, may serve as crucial biomarkers for the identification of preneoplastic lesions. Recent research has primarily focused on circulating nucleic acids, epigenetic alterations, proteomics, metabolomics, and parasite-specific molecules.

### MicroRNAs

MicroRNAs are considered strong candidate biomarkers for early detection. On the host side, multiple studies have found that miR-21 is consistently upregulated in the plasma of CCA patients, where it promotes CCA cell proliferation and migration and correlates with patient prognosis [15, 38, 77]. Additionally, other miRNAs such as miR-150 are also related to CCA risk [63]. Recent studies indicate that circulating and exosome-derived miRNAs, particularly exosomal miR-21, can be used for non-invasive early detection [39, 44]. However, these miRNAs or their homologs were not confirmed as a direct result of *C. sinensis* infection, but this suggests that miRNAs contained within exosomes of *C. sinensis* – such as Csi-let-7a-5p and Csi-miR-96-5p, may also act as risk molecular markers [51].

### DNA methylation and epigenetics

DNA methylation alterations are evident even in the early stages of parasite-associated CCA. Research using quantitative methylation-specific PCR (qMSP) has shown that specific methylation sites of four genes (*CDO1*, *CNRIP1*, *SEPT9*, and *VIM*) in biliary brush specimens can identify CCA with high sensitivity [2]. Other studies have proposed methylation panels capable of distinguishing HCC from CCA [4], providing a basis for early clinical subtyping. Epigenetic markers not only aid in early diagnosis, but may also illuminate carcinogenic mechanisms driven by chronic inflammation [6, 53].

### Proteomics and serological biomarkers

Proteomics-based studies have identified a cohort of candidate molecules induced by *C. sinensis* ESPs, specifically including:

1. Key signaling pathway proteins: specifically, molecules related to the “Integrin β4-FAK/Src” signaling axis, including focal adhesion kinase (FAK, phosphorylated at Y397 for activation), Src family kinases (Src, phosphorylated at Y419 for activation), and downstream effectors like paxillin (phosphorylated at Y118) and vinculin (phosphorylated at Y1065). These facilitate signal transduction and the connection between the cytoskeleton and the ECM [48].
2. Parasite-specific proteins: *C. sinensis* cathepsin B protein is closely associated with tissue damage during parasitism. This cathepsin B protein is encoded and expressed by *C. sinensis* itself and is natively present in specific tissues and secretory products of the parasite [11].
3. Metalloproteinases: Such as matrix metalloproteinases (MMP9, involved in ECM degradation). The molecules jointly promote the migration and invasion of CCA cells, with integrin β4, FAK, Src, paxillin, vinculin, and MMP9 acting as core regulatory nodes [48, 49, 53, 79].

Metabolomic studies have detected significant differences in serum metabolite profiles between infected and non-infected individuals, offering new avenues for early diagnosis [60]. Further serological studies have identified Omega-class GST as a reliable serological marker capable of discriminating clonorchiasis from opisthorchiasis [1, 29]. In host tissues, high-throughput proteomic methods have also identified several antigens suitable for serological detection [13], and differential proteomics has revealed candidate biomarkers in CCA tissue [27].

### Integrated biomarkers and clinical application prospects

Recent reviews suggest that early detection of CCA requires multi-level biomarker panels, incorporating miRNAs, DNA methylation markers, proteomic, and metabolomic data [41, 62]. The advancement of liquid biopsy technologies has made the detection of circulating DNA and exosomes feasible [44]. In the future, combining parasite-derived molecules with host molecular markers holds promise for enhancing the early screening capacity for *C. sinensis*-associated CCA and promoting personalized intervention (Table 1) [51, 53].

**Table 1:** Molecular products derived from *C. sinensis* infection and associated host response molecular biomarkers.

Category	Representative biomarkers/molecules	Study subject	Main findings	Sample size	Cohort type	Reference
miRNA, Exosomes	miR-21, miR-150, exosomal miR-21	Plasma, CCA Cells, EVs	miR-21 is upregulated, predicts prognosis, promotes tumor progression; EV-carried miRNAs participate in malignant transformation.	miR-21: *n* = 74 (74 ICC post resection)miR-150: *n* = 70 (35 CCA/35 controls)exosomal miR-21: *n* = 60 (30 CCA/30 controls)	miR-21: Retrospective case cohort study. miR-150: Retrospective case-control study. exosomal miR-21: Retrospective case-control study.	[15, 38, 44, 51, 64, 77]
DNA Methylation	Specific Loci	Biliary Brush Samples, Plasma DNA	Can distinguish CCA from non-cancer; some panels differentiate HCC from CCA.	Tissue Samples: *n* = 93 (39 CCA/54 controls)Biliary Brushing Samples: 103 samples from 92 patients.	Retrospective Study	[2, 4]
Proteomics	Differential Proteins, ESP-induced Proteins, Antigens	CCA Tissue, Serum, ESP	ESP proteins activate host cells; ω-GST serves as a serological marker; differential proteomics identifies candidates.	ω-class GST: *n* = 499 (207 liver fluke-infected/252 other parasitic infections/40 controls)	ω-class GST: Retrospective cohort study.	[1, 13, 29, 32, 49, 79]
Parasite-Specific Molecules	*Cs*Cathepsin B	Parasite Proteins, Host Tissue	Associated with tissue damage and biliary epithelial alterations.	–	Retrospective Pathological Sample Study	[11, 29]
Metabolomics	γ-glutamyl transpeptidase, D-glucuronic acid	Serum from Infected Individuals	Can distinguish infection status, suggests link between infection and carcinogenesis.	*n* = 30 (22 *C. sinensis*-infected cases/8 controls).	Retrospective Cohort Study	[60]

It summarizes potential molecular biomarkers from various categories including miRNAs, DNA methylation, proteins, parasite-specific molecules, and metabolites. Includes representative biomarkers, study subjects, key findings, sample sizes, study types, and references. “–” refers to “Not mentioned”.

### Challenges in the clinical translation of biomarkers

Despite considerable progress in the research of early molecular biomarkers for clonorchiasis, significant obstacles still remain in translating laboratory findings into routine clinical practice.

Technically, the standardization of detection methods is essential. Current studies employ diverse techniques and platforms for detecting biomarkers like miRNAs and DNA methylation, leading to a lack of comparability in results. For miRNA detection, variations in RNA extraction efficiency, reverse transcription, and quantitative PCR protocols can cause substantial discrepancies in measured miRNA expression levels. Similarly, in DNA methylation analysis, differences in methylation-specific PCR primer design and experimental conditions impact the accuracy and reproducibility of results [2]. Furthermore, proteomic and metabolomic studies lack uniform standards for sample preparation and mass spectrometry analysis, which limits the generalizability and application of their findings.

From an epidemiological perspective, most current studies are single-center investigations with small sample sizes, making it difficult to accurately reflect the true situation of *C. sinensis* infection and preneoplastic lesions across different regions and populations. Variations exist in prevalent *C. sinensis* strains across regions, and differences in population genetic backgrounds, lifestyle habits, and infection intensity all influence the expression and diagnostic efficacy of molecular biomarkers. In highly endemic areas, where infections are often long-standing and intense, biomarker profiles may differ from those in low-endemicity regions. Variations in immune responses and molecular reactions to *C. sinensis* infection among different ethnic groups may also limit the broad applicability of specific biomarkers [32, 87].

The cost of clinical testing is another major factor restricting the application of molecular biomarkers. Techniques like DNA methylation analysis, proteomics, and metabolomics typically require expensive instrumentation and specialized technical personnel, which results in high detection costs that hinder large-scale implementation in primary healthcare settings and endemic regions with limited resources. Additionally, these detection workflows are complex, requiring considerable time from sample collection and processing to result reporting, which fails to meet the demand for rapid clinical diagnosis. Moreover, clinical awareness and acceptance of these emerging molecular biomarkers need improvement, requiring enhanced training to enable healthcare providers to better understand and utilize these markers for disease diagnosis and management.

### Innovative technologies facilitating clinical translation

Emerging technologies show potential to address challenges related to high costs, standardization difficulties, and uneven resource distribution, and this will surely facilitate clinical translation. Isothermal amplification technologies like loop-mediated isothermal amplification (LAMP), known for their simple equipment requirements, rapid reaction times, and low cost, have been successfully applied in field screening for several parasitic diseases. They could be developed for the rapid detection of nucleic acid biomarkers related to *C. sinensis* infection and early carcinogenesis, making them suitable for primary care settings and field screening in endemic areas [34]. CRISPR-Cas systems combined with portable reading devices offer high sensitivity and specificity and are increasingly used for rapid molecular diagnosis. They hold future promise for point-of-care testing of *C. sinensis*-specific nucleic acids or host methylation markers in bile or blood [18]. Artificial intelligence (AI)-assisted image analysis can also improve the identification efficiency of preneoplastic lesions by training models to recognize early biliary abnormalities in ultrasound, computed tomography (CT), or magnetic resonance imaging (MRI) scans, reducing reliance on specialist physician experience. This is particularly suitable for promotion in resource-limited areas [76]. Technologies like microfluidic chips and paper-based sensors can integrate sample processing and detection steps, enabling “sample-in, answer-out” operation and providing portable, low-cost platforms for multi-marker detection. Future efforts should strengthen the adaptability research and multi-center validation of these technologies specifically for the early screening of clonorchiasis and CCA, promoting their translation from the laboratory to field application.

## Technologies and challenges in identifying preneoplastic lesions

Importantly, *C. sinensis*-associated preneoplastic lesions such as chronic cholangitis, biliary epithelial hyperplasia, and biliary intraepithelial neoplasia often have an insidious onset and lack specific symptoms, posing immense challenges for early clinical identification. With advancements in imaging, endoscopic techniques, molecular diagnostics, and multi-omics, various methods are being applied to detect and assess preneoplastic lesions, yet each still faces notable limitations.

### Imaging and cholangioscopy techniques

Conventional imaging (ultrasound, CT, MRI) is primarily used clinically to detect biliary strictures or intrahepatic space-occupying lesions. While highly sensitive for advanced CCA, their ability to identify preneoplastic lesions is limited. Conventional transabdominal ultrasound has a low sensitivity for early-stage CCA, and often reveals only non-specific inflammation or mild strictures [43, 52, 61]. Consequently, these shortages have promoted the exploration of endoscopic techniques for direct visualization and targeted sampling.

Endoscopic retrograde cholangiopancreatography (ERCP) is a kind of endoscopy. ERCP with brushing and biopsy are the most useful histological methods. Brushing alone has a sensitivity of less than 50% [17, 47], and biopsy alone is prone to false negatives due to insufficient tissue sampling or tumor heterogeneity. Cytological brushing remains widely used due to its procedural simplicity, but its sensitivity is limited by tumor cell exfoliation rates and pathological expertise, leading to frequent false negatives. The “triple sampling” method combining brushing, biopsy, and fluorescence *in situ* hybridization (FISH) improves sensitivity, but remains insufficient for the early detection of preneoplastic lesions [36, 46]. While the integration of imaging and ERCP has significantly improved the diagnostic accuracy for advanced disease, early stage lesions often remain undetected, motivating the introduction of digital cholangioscopy.

Advancements in cholangioscopy, such as digital single-operator cholangioscopy (DSOC), enable direct visual-guided biopsy, significantly improving the identification rate of atypical strictures and early lesions [63, 69]. The comprehensive sensitivity of DSOC for indeterminate biliary strictures reaches 74%, significantly higher than conventional ultrasound/CT [82]. International consensus also recommends cholangioscopy as an important tool for managing difficult biliary strictures [3]. However, this method is limited by expensive equipment, high technical skill requirements, small sample sizes, and lack of procedural standardization, which hinders its widespread adoption in endemic areas. Also, morphological assessment is unable to capture molecular-level changes, thus highlighting the demand for molecular diagnostic approaches.

### Molecular diagnostics and liquid biopsy

Advances in molecular biology have provided new opportunities for early detection of preneoplastic lesions using molecular markers in bile and brushing samples.

DNA Methylation Detection: Multiple studies confirm the presence of specific methylation markers in biliary brushings and bile samples that can identify CCA with high sensitivity, with some detectable even at the preneoplastic stage [2, 74, 89]. In ERCP brushing samples, a panel of methylation markers like CDO1 and SEPT9 demonstrated sensitivity up to 85% for detecting CCA with high specificity [2, 74]. Combining methylation detection with conventional ERCP brushing cytology, serum markers like CA19-9, or imaging can significantly increase overall sensitivity; studies combining methylation indices/multiple tests have reported sensitivities approaching 90–97% [55]. These findings suggest DNA methylation detection can powerfully complement cytology and fill the blind spots of morphological detection. However, its clinical promotion is limited by detection costs and laboratory standardization issues.

**Figure 5:**
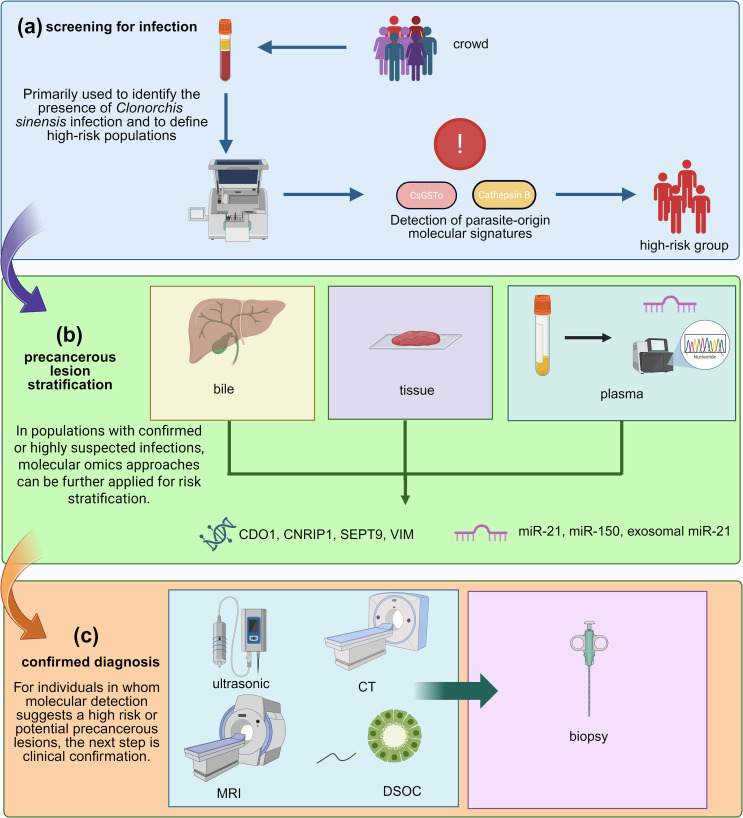
Workflow of integrated multi-omics biomarkers. It outlines the systematic pipeline from sample collection and multi-omics data analysis such as transcriptomics, proteomics, metabolomics, exosomes to biomarker validation and clinical application. *Cs*GSTo: *Clonorchis sinensis* Glutathione S-Transferase omega, CDO1: Cysteine Dioxygenase Type 1, CNRIP1: Cannabinoid Receptor Interacting Protein 1, SEPT9: Septin 9, VIM: Vimentin, miR-21: microRNA-21, miR-150: microRNA-150, CT: Computed Tomography, MRI: Magnetic Resonance Imaging, DSOC: Digital Single-Operator Cholangioscopy.

miRNA and Exosomes: miRNAs and exosome-related molecules in bile and plasma show potential for differentiating CCA from preneoplastic lesions [5, 37]. These two types of biomarkers are mutually complementary – miRNAs indicate dynamic inflammatory responses and microenvironmental remodeling, while DNA methylation captures more stable epigenetic modifications, which indicates multi-omics profiling may provide superior diagnostic accuracy compared with individual biomarkers. However, as mentioned above, miRNA detection may be unstable, these markers also lack validation in large-scale cohorts (Fig. 5).

Circulating Tumor DNA (ctDNA): Liquid biopsy has made plasma ctDNA a research focus. Although preliminary results show promise for early cancer detection, its sensitivity and specificity for the inflammatory and preneoplastic stages remain inadequately defined [7].

### Proteomics and metabolomics

Proteomic studies have identified a series of differentially expressed molecules. For instance, LC-MS proteomics has revealed candidate biomarkers in CCA tissue [49], and bile proteome analysis has also identified various potential molecules for early diagnosis [67].

Metabolomics similarly indicates that bile and serum metabolite profiles can distinguish between infection, inflammation, and early cancerous states [5]. We can propose that the integration of proteomics and metabolomics will greatly enhance the diagnostic robustness. The former reveals the underlying molecular mechanisms, whereas the latter reflects systemic physiological responses. However, limitations such as small sample sizes and insufficient reproducibility keep these findings primarily at the research stage.

## Intervention strategies and future perspectives

The control of clonorchiasis has historically relied primarily on drug therapy, notably PZQ. A regimen of 25 mg/kg three times daily for 2 days demonstrates high efficacy against adult worms and a favorable safety profile, making it widely used for both individual treatment and mass drug administration (MDA) [9]. However, the high reinfection rate, potential risk of drug resistance, and the inherent limitation of chemotherapy alone in interrupting transmission highlight the constraints of this traditional approach [21, 86]. MDA programs and targeted chemotherapy pilots in endemic areas have shown that infection rates readily rebound unless combined with health education and environmental management [12, 24]. Therefore, a key future challenge lies in promoting sustainable control while maintaining drug accessibility.

Recent modeling and health economics research provide a basis for formulating precise control strategies. Analyses based on dynamical models indicate that the effectiveness of drug intervention alone is limited across different transmission intensities, whereas integrated interventions (combining drug therapy, health education, and food-safety control) demonstrate long-term cost-effectiveness [20, 22, 59]. This indicates that future policies should focus more on designing combined interventions tailored to specific population contexts, moving away from a one-size-fits-all MDA model.

In diagnostics, the insufficient sensitivity of traditional stool examination and imaging for early detection limits their effectiveness for population screening and recurrence monitoring. Emerging serological biomarkers [29] and rapid molecular tests [40] hold promise for widespread application in primary care or endemic settings, potentially enhancing screening efficiency. However, validation of these methods across different regions and populations with varying infection intensities remains limited, urgently requiring larger multi-center studies to facilitate their translation. To address this gap, future studies need to launch a multicenter prospective cohort (*n* > 1000) across *C. sinensis*-endemic areas to validate the diagnostic value of the potential biomarkers, aiming to establish a standardized detection protocol.

A more prospective direction involves vaccine development. Although no approved vaccine against *C. sinensis* currently exists, several candidate antigens such as enolase, paramyosin, cysteine proteinase have shown different degrees of immunoprotective efficacy in animal models [35, 71, 72]. Oral vaccines based on Bacillus subtilis spore delivery vectors are particularly noteworthy. This oral vaccine effectively induced mucosal immune responses in animal models. In mice orally administered 5 × 10^8^ CFU for 5 consecutive days with a booster on day 21, increased serum IgG was observed at both day 21 and 42 (approximately 2-fold higher than controls at day 42), alongside significant increases in IgA detected in bronchoalveolar lavage fluid and feces (*p* < 0.001), although no difference was seen in salivary IgA. In a pig model, oral administration of 10^7^ CFU/kg also significantly elevated serum anti-GP5 IgG levels and generated neutralizing antibodies against EU-type and NA-type PRRSV by day 42; concurrently, IgA levels in saliva and feces were markedly increased (*p* < 0.001), accompanied by upregulation of IL-4 and IFN-γ, indicating its ability to stimulate mucosal IgA, humoral, and cellular immune responses [45]. This approach not only induces mucosal immunity in experimental animals but also possesses potential advantages for population-level application [71]. Future efforts combining molecular adjuvants, delivery systems, and cross-protection studies could position vaccination as a long-term strategy capable of transforming disease control.

From a global perspective, clonorchiasis control is intricately linked to food safety, cultural practices, and public health systems [58, 73]. Single technical approaches are often insufficient for sustained effectiveness. Therefore, the future direction lies in adopting a One Health framework which integrates human, animal host, and environmental aspects and fostering cross-sectoral collaboration. Furthermore, the emerging application of AI analytics and big data in epidemiological prediction and precision intervention holds potential for driving more efficient control in resource-limited settings [8].

Future intervention strategies should be grounded in integrated management and technological innovation: relying short-term on combining chemotherapy with health education, developing mid-term precision surveillance systems based on molecular and rapid field diagnostics, and promoting long-term vaccine development alongside environmental and food-borne risk control. Only through multi-level, multi-sectoral collaboration can the transition be made from merely reducing infection rates towards reducing disease burden and ultimately elimination.

## Conclusion

Significant progress has been made in research on early molecular biomarkers and the identification of preneoplastic lesions in clonorchiasis, yet numerous challenges persist. Multi-omics technologies have elucidated the molecular characteristics of *C. sinensis* infection and its carcinogenic mechanisms, uncovering a plethora of potential diagnostic biomarkers. However, the clinical translation of these biomarkers faces a bottleneck, hindered by a lack of multi-center validation and standardized protocols. Concurrently, resource allocation issues present a conflict, namely the conflict between high-cost technologies and the scarcity of medical resources in endemic areas. Advances in imaging technology and artificial intelligence have improved the capacity to identify preneoplastic lesions, but cost-effectiveness and accessibility remain significant challenges in resource-limited settings.

Future research ought to probe deeper into the carcinogenic mechanisms of *C. sinensis* infection, develop novel detection technologies, conduct large-scale cohort validations, and evaluate intervention effectiveness. This should be coupled with integrating parasitology, oncology, and bioinformatics to advance precision diagnosis. The ultimate goal is the early prevention and control of *C. sinensis*-associated cancer. Multidisciplinary collaboration and translational research will be pivotal to achieving this objective.
